# Refinement and prediction of protein prenylation motifs

**DOI:** 10.1186/gb-2005-6-6-r55

**Published:** 2005-05-27

**Authors:** Sebastian Maurer-Stroh, Frank Eisenhaber

**Affiliations:** 1 IMP - Research Institute of Molecular Pathology, Dr. Bohr-Gasse 7, A-1030 Vienna, Austria

## Abstract

We refined the motifs for carboxy-terminal protein prenylation by analysis of known substrates for farnesyltransferase (FT), geranylgeranyltransferase I (GGT1) and geranylgeranyltransferase II (GGT2). In addition to the CaaX box for the first two enzymes, we identify a preceding linker region that appears constrained in physicochemical properties, requiring small or flexible, preferably hydrophilic, amino acids. Predictors were constructed on the basis of sequence and physical property profiles, including interpositional correlations, and are available as the Prenylation Prediction Suite (PrePS, ) which also allows evaluation of evolutionary motif conservation. PrePS can predict partially overlapping substrate specificities, which is of medical importance in the case of understanding cellular action of FT inhibitors as anticancer and anti-parasite agents.

## Abstract 2

Three prenylation motif predictors are presented that allow discrimination between proteins that are unique substrates of farnesyltransferase (FT) and those that can be alternatively processed by geranylgeranyltransferase I (GGT1).

## Rationale

Prenylation refers to the posttranslational modification of proteins with isoprenyl anchors [1-3]. These lipid moieties are typically involved in mediating not only protein-membrane but also protein-protein interactions. Three eukaryotic enzymes are known to catalyze the lipid transfer. The first two, farnesyltransferase (FT) and geranylgeranyltransferase 1 (GGT1), recognize the so-called CaaX box in the carboxy termini of substrate proteins and attach farnesyl (15-carbon polyisoprene) or geranylgeranyl (20-carbon polyisoprene), respectively, to a required and spatially fixed cysteine in that motif. The third enzyme, geranylgeranyltransferase 2 (GGT2 or RabGGT) recognizes the complex [4] of Rab GTPase substrate proteins with a specific Rab escort protein (REP) to attach one or two geranylgeranyl anchors to cysteines in a more flexible but also carboxy-terminal motif.

The CaaX box was initially understood to consist of a cysteine (C), followed by two aliphatic residues (aa) and a terminal residue (X) that would direct modification by either FT or GGT1, but newly found substrates and kinetic studies of mutated substrate peptides and enzyme inhibitors have shown that the motif recognized by the enzymes appears to be more flexible [2]. Furthermore, the determination of preference for FT or GGT1 is more complex and a function of the overall sequence context rather than specific amino acids at single positions. Whereas GGT2 appears to be specific to Rab GTPases as substrates, the recognition mechanism is not well understood. Overlapping substrate specificities between all three prenylating enzymes further complicate the understanding of the lipid modification process [5,6].

An unsolved problem so far is accounting for the complexity of the prenylation substrate recognition motifs in theoretical models in order to identify substrate proteins from their amino-acid sequence. No available method has been able to selectively assign the correct modifying enzyme, which determines the types and number of lipid anchors. The high probability of motifs similar to the small CaaX box occurring by chance is a general problem that has so far prohibited large-scale proteome analyses [7]. We describe here a method that aims to model the substrate-enzyme interactions on the basis of refinement of the recognition motifs for each of the prenyltransferases. The Prenylation Prediction Suite (PrePS) selectively assigns the modifying enzyme to predicted substrate proteins and sensitively filters out false-positive predictions based on the general methodology that has already been applied successfully for the prediction of glycosylphosphatidylinositol (GPI) anchors [8], myristoylation [9] and PTS1 peroxisomal targeting [10].

## Known substrates and their motif-compliant homologs as learning sets

The first task consists of collecting sequences that are known substrates for the respective enzymes. Typically, a good starting point is the Swiss-Prot database [11]. However, according to earlier experience with annotation inaccuracies [12], any annotated experimental evidence has to be confirmed by following up all the related literature sources. As newly available data can be missing in the Swiss-Prot annotation, the searches have also to be extended to non-Swiss-Prot proteins. In most cases, the annotations for prenylation in Swiss-Prot are assigned by similarity to only a few entries with experimental validation. A major concern is the annotation of the correct anchor type attached to FT and GGT1 substrates, which could previously only tentatively be estimated without experimental data. This includes several entries with overall sequence similarity to a verified prenylated protein but totally different carboxy-terminal motifs. Given that single mutations can abolish recognition or switch enzyme specificities [13] and that not all homologs of lipid-modified proteins necessarily have to share the same modification type or membrane attachment factor (MAF) [14], entries with annotations only by similarity should not be included without critical consideration in a learning set.

Unfortunately, such justified concerns dramatically lower the amount of data in the learning set. However, because of earlier interest in developing peptide-based inhibitors of FT and GGT1 as anticancer treatments, the kinetics of the enzymes with various tetrapeptide substrates already modified with lipid anchors by the enzymes have been measured [15]. Hence, a protein homologous to a verified prenylated protein can be included in the learning set if its CaaX box has already been shown to interact productively with one of the prenyltransferases at least as a tetrapeptide.

However, possession of a valid CaaX box might not be a sufficient selection criterion. Typically, short terminal sequence motifs are connected to the rest of the protein by a linker region that experiences only limited constraints on specific amino acids per position but often has a compositional bias towards small or hydrophilic amino acids in connecting sequence stretches [16]. This property is found in a preliminary assembly of verified FT and GGT1 substrates and has been confirmed in the actual learning set for up to 11 residues upstream (amino-terminal) of the cysteine in the CaaX box (see below). Hence, learning-set sequences should also not violate the physicochemical properties constraining the sequence stretch amino-terminal to the CaaX box.

Taking account of the considerations above, the following procedure has been applied to obtain conservative and reliable learning sets of FT and GGT1 substrates. First, a literature search for known prenylated proteins and valid tetrapeptides (see [17]). Second, BLASTP [18] with an E-value threshold of 0.005 starting with known prenylated proteins against the National Center for Biotechnology Information (NCBI) nonredundant database to find homologs and cluster all collected sequences into groups of homologous proteins using the Markov-chain clustering algorithm (MCL) [19]. Third, check the validity of all CaaX boxes with experimental evidence for at least tetrapeptides. Fourth, check compliance with the physical properties of the full motif (including linker) by applying a preliminary predictor based on corrected Swiss-Prot entries in a similar style as described here (penalizing deviations from the physical property landscape of the motif).

This resulted in learning sets of 692 FT and 486 GGT1 substrates, respectively (see [17]). Among the FT substrates, 31 artificial constructs or mutations of naturally occurring sequences that have been shown to be processed by FT have also been included. Prenylation by GGT2 follows totally different mechanistic requirements than FT and GGT1 and will be treated separately after the sections about CaaX prenylation.

## Refinement of the CaaX box motif descriptions

**Figure 1:**
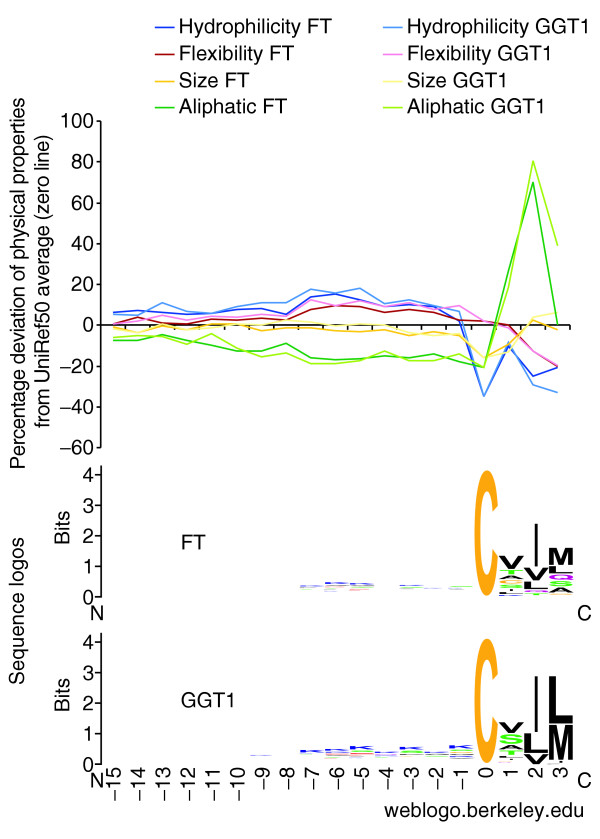
Sequence logos [74] and physicochemical property profiles of FT and GGT1 substrates. Selected physical properties (hydrophilicity = KRIW790102; flexibility = KARP850103, size = CHOC760101; aliphatic = ZVEL_ALI_1; see Tables 1 and 2 for details) are calculated as average over the nonredundant learning sets of FT and GGT1. The plotted lines correspond to the relative deviation of the respective properties from an average calculated over carboxy termini from the UniRef50 database [22].

Compositional analysis of residue frequencies at single motif positions reveals that major restrictions to specific amino acid types exist only for positions within the CaaX box (see sequence logos in Figure 1). The previously reported preferences for aliphatic residues at positions +1 and +2 (the aa in CaaX) were recovered, but there is a clear tendency for other residue types to also be allowed, especially at position +1 (the first a in CaaX). Correlation analysis of residue frequencies at single motif positions with amino-acid property scales [20,21] can quantify the conservation of a physical property pattern (see Materials and methods). Although correlations higher than 0.6 can only be obtained for aliphatic property at position +2 (FT: 0.85, GGT1: 0.87), the average aliphatic property at position +1 within both FT and GGT1 learning sets still appears elevated when compared to an average calculated from the carboxy-termini of the nonredundant UniRef50 database [22] (see physical property profile in Figure 1). Similarly, there are correlations at position +2 and deviations from the UniRef50 average at position +1 for a property describing preference for extended conformations (see Tables 1 and 2). This appears to be best explained by the need to have the final peptide part in extended conformation rather than coiled or helical in order to fit into the binding pocket, as can be seen in the resolved structures of prenyltransferases with their substrate peptides [23].

**Figure 2:**
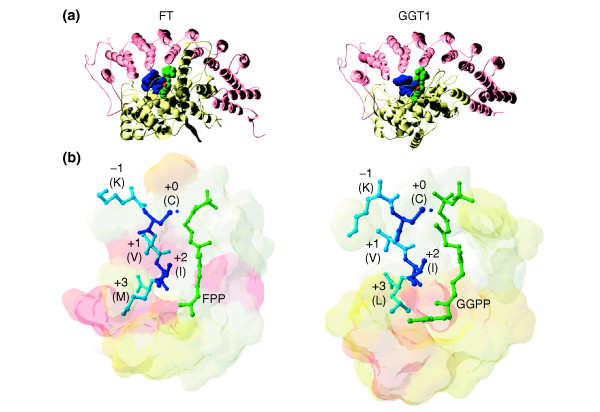
The two CaaX prenyltransferases. **(a) **Ribbon representations of FT (PDB 1D8D [75]) and GGT1 (PDB 1N4Q [76]); pink, alpha subunit; yellow, beta subunit. **(b) **The prenylpyrophosphates (green) and CaaX tetrapeptides (blue) inside the binding pockets with enzyme-specific conservation (conservation in FT or GGT1 minus conservation in joined FT+GGT1 alignment) mapped to binding-pocket surface. Increasing conservation difference is shaded from white to yellow to red. FPP, farnesyl-, GGPP, geranylgeranylpyrophosphate. The alignment of the sequences of these proteins is shown in Figure 6. Visualized with Swiss-Pdb Viewer [59].

The major difference between FT and GGT1 substrates remains at position +3 (the X in CaaX). Whereas a broad variety of residues are allowed in motifs recognized by FT (including several substrates with leucine at +3), mainly leucine and methionine appear to be preferred by GGT1 in agreement with experimental evidence [13]. Interestingly, position +3 correlates (FT, 0.7; GGT1, 0.8) with a physical property that measures membrane-buried preference parameters (see Tables 1 and 2). This feature does not seem to be important to support membrane interaction at a later stage for the protein, as the three carboxy-terminal residues (-aaX) are often cleaved off in a further processing step after attachment of the anchor [24]. However, hydrophobicity and volume of position +3 appear important for interaction with the binding pocket because of the rather lipophilic character of the latter (isoprenyl anchor on one side and hydrophobic residues on the others). The importance of position +3 for specificity between FT and GGT1 is further strengthened by differing conservation of residues in the binding pockets of the respective enzymes (Figure 2). Not surprisingly, the whole region of the binding pocket harboring the end of the prenylpyrophosphate (geranylgeranyl [C20] is one isoprene unit longer than farnesyl [C15]) and the X of the CaaX box (position +3) appear to comprise the major differences in residue conservation (Figure 2).

Using the Fisher criterion (see Materials and methods), interpositional correlations of residue sizes within positions +1, +2 and +3 (the carboxy-terminal three residues of the CaaX box that are buried in the binding pocket) from both FT and GGT1 substrates have been identified. Often, when a very large residue occurs at specific positions, neighboring residues compensate to obey the overall physicochemical constraints (for example, size limitation) in the binding pocket. Similarly, compensatory effects appear to exist regarding hydrophobicity between positions +1 and +3 in FT and between +1, +2 and +3 in GGT1 substrates (see Tables 1 and 2). Compensatory effects also seem responsible for the toleration of even large positively charged residues at positions +1 or +2, if the other residues are small enough to accommodate the whole peptide in the binding pocket. On the other hand, negative charges are apparently incompatible with the substrate recognition motif at these positions.

## Extension of the CaaX prenylation motif by a flexible linker region

While the requirement for specific amino acids at single positions appears to be marginal outside of the CaaX box, physicochemical constraints that extend up to 11 residues amino-terminal from the modified cysteine can be found (Figure 1, Tables 1 and 2). At position -1 of the motif, there begins a pronounced tendency for residues with either small or flexible hydrophilic side chains. GGT1 especially appears to prefer amino acids like serine or lysine at this position. In general, GGT1 substrates have a higher number of lysines within positions -1 and -7 compared with the FT substrates.

The hydrophilic linker region with correlations over multiple positions to several hydrophobicity- and flexibility-related property scales might be required to allow accessibility of the carboxy terminus for the lipid-attaching enzymes. Indeed, in several resolved structures of *in vivo *prenylated GTPases, secondary structural elements such as helices that stabilize the fold of the protein are typically found only at the amino-terminal side of that linker region (beginning of helix at positions -12 (PDB identifier 1FTN), -13 (PDB 1MH1), -15 (PDB 1AM4), -12 (PDB 1A4R)). In the structure of a G protein gamma subunit, the linker region also appears to be extended and wrapped around the beta subunit in the heterotrimeric G protein signaling complex (PDB 1GG2). It needs to be emphasized that the linker region must not necessarily be in an unstructured conformation after the anchor has been attached (see also carboxy-terminal helix in structure PDB 1F5N of human 67 kDa guanylate binding protein 1 [25]), as folding back or lipid-mediated interaction with other proteins or membranes can also induce changes in the three-dimensional structure of the linker region. However, there appears to be a requirement for the ability to easily unfold/fold into flexible and more extended conformations that allow the carboxy terminus to be accessed and modified by the prenyltransferases. It is noteworthy that this length estimation of a flexible, hydrophilic linker is consistent with earlier findings in the GPI anchor [21], myristoylation [12] and PTS1 targeting [26] motifs. Hence, the actual motif length of substrates for CaaX prenylation appears longer than previously thought (total 15 residues = 4 CaaX + 11 linker).

## Prediction function and validation

Following the approach already applied to the prediction of GPI and myristoyl anchors and PTS1-mediated targeting [8-10], a scoring function measuring compliance with the prenylation motif separately for the enzymes FT and GGT1, respectively, has been constructed (see Materials and methods). In brief, the composite prediction function *S *consists of a term *S*_profile _scoring a query sequence against the redundancy-corrected profile of the learning-set sequences and another term *S*_ppt _that penalizes deviation from the physicochemical motif requirements.

***S ***= ***S***_profile _+ ***S***_ppt_

The term *S*_profile _distinguishes the three positions +1, +2 and +3 of the CaaX box as well as the linker region (-1 to -11). *S*_ppt _comprises a sum of terms that are constructed from the physical property requirements for FT and GGT1 substrates that were outlined in the section describing the motif refinement (and listed in Tables 1 and 2 together with their rationale for inclusion in *S*_ppt_).

The threshold for a query protein to be a predicted farnesylation or geranylgeranylation target by FT or GGT1, respectively, is set to include all sequences in the learning set. Hence, the self-consistencies or upper bounds of sensitivities of the FT and GGT1 predictors are 100%. Additionally, the robustness of the method has been cross-validated in jackknife tests (see Materials and methods). In the cross-validation over the complete scoring function, the rates of finding known substrates after excluding them and their close homologs from the learning procedure (and, therefore, lower bounds for sensitivities) were 92.6% for FT and 98.6% for GGT1, respectively.

As required for a good predictor [16], the scores are translated into probabilities of false-positive prediction. For this purpose, a sigmoidal function (analytically based on the extreme-value distribution) is fitted to the distribution of score values calculated from non-prenylatable proteins (see Materials and methods). The general probabilities of false-positive prediction (that complement the specificities to 100%) are estimated to be 0.11% for the FT and 0.02% for the GGT1 predictor, respectively.

### Capability to distinguish FT and GGT1 substrates

Previously, the assignment of CaaX box substrate proteins to either FT or GGT1 has been based mainly on the identity of the final residue in the motif (position +3) where FT allows several amino-acid types and GGT1 clearly prefers leucine [13,27]. This view has not changed but it has become clear that several substrates with leucine at position +3 can also be modified (if only to a lesser extent) by FT and not only GGT1. For example, *in vitro *studies have shown that motifs like CVIL, CVLL, CAIL and CCIL (single-letter amino-acid code) are valid for FT as well [28]. Mutation of the CVIA motif of yeast A-factor to CVIL results in geranylgeranylated as well as farnesylated proteins *in vivo *[29]. Also, RhoB (with a CKVL motif) is known to be both farnesylated and geranylgeranylated *in vivo *[30]. Similarily, substrate proteins ending with phenylalanine, such as the CVIF of R-Ras2/TC21, are not specific to either enzyme and can be substrates to FT and GGT1 [31].

In the same way that FT can accept CaaX box motifs ending in leucine and phenylalanine, GGT1 appears to tolerate methionine at this position, which was previously thought to direct farnesylation. This has important consequences in the case of the oncoprotein K-Ras (in variants with CVIM and CIIM motifs) which becomes geranylgeranylated *in vivo *when farnesyltransferase is inhibited [32].

**Figure 3:**
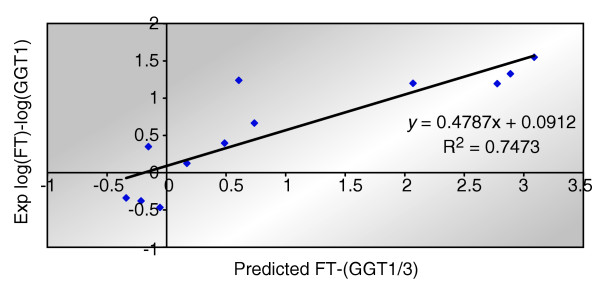
Correlation between predicted and experimental FT/GGT1 substrate selectivity. The correlation of the difference between predicted FT and GGT1 scores with the difference of the experimentally measured logarithmic affinities for FT and GGT1 of the same substrates is plotted.

As we have experienced with our earlier predictors for myristoylation and PTS1 targeting, we find even some correlations of the prediction scores with experimentally measured substrate-enzyme affinities. Interestingly, the scores of the GGT1 predictor give better agreement with the experimental data when divided by 3, in agreement with a threefold lower *in vivo *activity of GGT1 compared to FT [5]. To estimate the capability of the FT and GGT1 predictors to model the overlapping but distinct substrate specificities, we analyzed a set of heterogeneous substrate motifs that have been measured under the same experimental conditions for their affinities to either FT or GGT1 [5] and we tried to correlate these experimental data with our prediction scores. The set of motifs (CVLS, CIIS, CIIC, CVLF, CVIM, CAIM, CAIV, CAII, CAIL, CVVL, CIIL, and CTIL) contains a large fraction of examples that have been previously shown to be cross-reactive between FT and GGT1 or where the assignment based on simple heuristics depending on hydrophobicity of the final residue fails. In Figure 3, we have plotted the difference of predicted FT and GGT1 scores against the difference of experimentally measured logarithmic affinities for FT and GGT1. A correlation of 0.74 indicates that the theoretical interaction model implemented in the prediction function at least semi-quantitatively resembles the relative substrate specificities between FT and GGT1.

### Prediction of prenylation by GGT2

**Figure 4:**
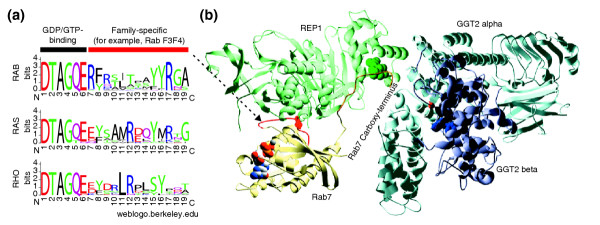
Determinants of GGT2 prenylation. **(a) **Sequence logos [74] of Ras superfamily members around part of the Rab-REP interaction site (colored red in the otherwise yellow GTPase structure). **(b) **Structural model of the Rab-REP-GGT2 prenylation complex based on PDB entries 1LTX [77] and 1VG0 [4]. REP1 (green) has a prenyl-binding pocket which is proposed to be involved in the dual geranylgeranylation mechanism (bound geranylgeranyl is shown in green). However, the catalytic attachment to the substrate cysteines takes place in the center of the GGT2 alpha-beta complex (light and dark blue) where the prenylpyrophosphate that will be transferred is also bound (blue space-filling representation, zinc in red). The structure was visualized using Swiss-Pdb Viewer [59].

Unlike FT and GGT1, substrate recognition by GGT2 is less dependent on strictly defined carboxy-terminal motifs, but on the complex formation of the substrate with an escort protein [4]. As illustrated in Figure 4, the substrate-escort protein complex then binds to GGT2 (consisting of the alpha and beta subunit typical of prenyltransferases) and, thereby, positioning the flexible substrate carboxy terminus towards the site of modification. Typically, the carboxy-terminal arrangement of cysteines is -XXXCC, -XXCXC, -XXCCX, -XCCXX or -CCXXX and, if available, both cysteines in such a motif will be geranylgeranylated. Currently, only the prenylation of Rab GTPases [33] with the help of Rab escort proteins (REP; two copies in higher organisms, otherwise only one copy) is known for the enzyme GGT2 which is, therefore, also called Rab geranylgeranyltransferase. Reports of lipid modification of fungal casein kinase I apparently represent carboxy-terminal palmitoylation [34] rather than the earlier postulated GGT2 prenylation [35].

Rab proteins are small GTPases (around 60 different have been identified in humans) [36] that share the general fold of the Ras superfamily as well as conserved residues in the nucleotide-binding site. Distinct motifs have been identified that are specific to the Ras, Rho, or Rab families [37]. By virtue of contributing to the binding site of Rabs with their REP, the Rab-specific F3F4 motif can be indirectly used to distinguish possible GGT2 substrates within the Ras superfamily (see sequence logos in Figure 4). However, the REP interaction motif (Rab F3F4) alone could be too short (13 residues) to allow highly sensitive large-scale database scans with thresholds that recognize the learning set (100% self-consistency requires a bit score greater than 5). Interestingly, a search with the final predictor against NCBI's nonredundant database finds only 34 hits with the F3F4 region alone that do not represent Rab proteins or their folds. To avoid these false positives, the hit to the overall alignment of Rab proteins with HMMer [38] (E-value < 0.1) is applied as additional prediction criterion to simulate recognition of the correct fold of related sequences.

Two alignments (F3F4 region and full length) were therefore constructed and after removal of entries with a maximal redundancy of 90% identity over the whole sequence length (117 of 179 entries annotated in Swiss-Prot remaining), hidden Markov models (HMMs) were created and calibrated. The choice of this methodology for the GGT2 prediction was strongly influenced by the fact that the HMMer [38] algorithm is well established in conservatively detecting fold homologies for globular domains at the sequence level. The final GGT2 prediction algorithm checks the carboxy termini for cysteines (at least one cysteine among the five last residues) and parses the HMMer outputs to combine the searches for final results. Estimates of false-positive prediction can be derived from the HMMer E-values.

## PrePS: Webinterface and EvOluation

**Figure 5:**
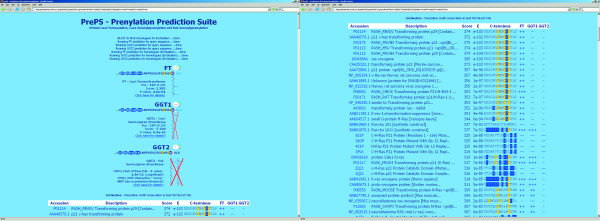
Screenshot of the output provided by the PrePS server [39]. On the left is the prediction result for the query protein H-Ras (GenBank P01112) and the three prenylating enzymes. On the right, is shown the carboxy-terminal alignment and PrePS predictions of homologs of the query protein for evaluation of evolutionary motif conservation. Note that H-Ras is predicted to be prenylated only by FT, whereas the homologs K-Ras and N-Ras can also be prenylated by GGT1.

The three tools to predict lipid modification by FT, GGT1 and GGT2 are available as Prenylation Prediction Suite (PrePS), which is accessible online [39]. Users can submit their query sequences to all three or selections of the single predictors. Details of the profile and physical property terms of the scoring function are provided and can also be used to check and rationalize whether and why certain query sequences or artificial constructs intended for membrane targeting might be less suitable prenylation targets. Additionally, an option is provided that allows the user to retrieve homologs of the query protein from NCBI's nonredundant database using BLASTP and automatically annotates them with their respective PrePS results. From the scores for the different predictors (left screenshot in Figure 5) as well as the alignment of the carboxy termini of homologous sequences (right screenshot in Figure 5), the evolutionary motif conservation can be evaluated (evOluation) and used for further rationalization of the biological importance of the predicted motif.

## Comparison with alternative methods

Until now, the only available tool to predict protein prenylation has been the Prosite [40] search with the pattern PS00294, which is also used in the PSORT II software [41]. However, this method can neither predict prenylation by GGT2 nor can it distinguish between modifications by FT or GGT1 and, hence, the attached anchor type. During preparation of this paper, an excellent study by Beese, Casey and colleagues [23] has been published that tries to define rules for substrate selectivity by crystallographic analysis of FT and GGT1 complexed with eight cross-reactive substrates. These detailed descriptions of the binding-pocket interactions of a few selected substrate peptides are in good agreement with the motif characteristics identified in this work. While the information gathered from the structural analysis exceeds the capability of any other purely theoretical method to judge interaction for the specific resolved enzyme-substrate pairs, it is difficult to generalize an interaction model from such a small dataset only on the basis of amino-acid constraints at single motif positions. Hence, applying these rules to a more restrictive Prosite-style pattern fails to identify around 30% of substrates experimentally verified in tetrapeptide interaction assays. When taking a closer look at known substrates that are not recognized by the rules of Beese, Casey and colleagues [23] it becomes apparent that this is mainly due to only a few factors. These are the exclusion of leucine at position +3 for alternative FT substrates (known example CKVL of RhoB), the exclusion of phenylalanine at position +3 for alternative FT substrates (known example CVIF of R-Ras2/TC21), the exclusion of glutamine at position +2 for FT substrates (known example serine/threonine kinase 11 or LKB1 with the motif CKQQ) and the exclusion of methionine at position +3 for alternative GGT1 substrates (known example CVIM of K-Ras). In addition, the rules of Beese, Casey and colleagues [23] assign isoleucine and valine at position +3 to GGT1 but not FT substrates. However, these two amino acids were shown to be valid for both FT and GGT1, with at least comparable affinities [13].

The inadequacy of the Beese, Casey and colleagues [23] motif in finding true-positive examples could be counteracted by loosening the motif description, as is already the case in the original Prosite entry PS00294, which nevertheless fails to predict known substrates with glutamine (LKB1) or proline (hepatitis delta antigen) at position +2. However, any reduction in motif stringency concomitantly results in a dramatic increase in the number of false-positive predictions. Table 3 compares typical prediction parameters for the different methods, if applicable. Neither the old nor an adjusted Prosite pattern can compete with the performance of PrePS in finding true substrates while, at the same time, only having a minimal number of false positives. The short Prosite patterns also do not take into account the linker region preceding the CaaX box, which is not defined by clear amino-acid type preferences but rather by general physicochemical property restrictions. The answers of Prosite-style predictions are only binary (yes/no), whereas PrePS gives continuous scores that can be split into interpretable motif-region contributions and that are shown to correlate with experimentally measured relative substrate affinities for FT or GGT1, respectively. Furthermore, only PrePS includes prediction of prenylation by GGT2 and provides an evaluation of evolutionary conservation of the prenylation motif among homologs of the query sequence.

## Medical implications and prediction examples

Farnesyltransferase inhibitors (FTIs) have been developed to prevent prenylation of oncogenic Ras proteins and are currently undergoing phase II and III clinical trials [42]. While FTIs have been suggested also to target parasitic diseases [24,43], their efficacy as cancer treatments has been found to be ambivalent in respect of different cancer types. This could be due to the alternative prenylation of oncogenic proteins by GGT1 under FT inhibition, such as K-Ras, in contrast to the total inhibition of prenylation for unique FT substrates, such as H-Ras [2,44]. Identifying these two types of substrate behavior is critical for understanding FTI action as well as identifying their real cellular targets [45,46]. One of the applications of PrePS is in the distinction of substrates that are specific to FT (FTI target) or GGT1 or that are modified by both (less affected by FTIs).

We would like to mention here one example prediction of PrePS for a protein that would be a candidate for a previously unknown FTI target. The human nucleosome assembly protein I-like protein [47] (NAP1-like (GenBank:NP_004528)) has a CKQQ farnesylation motif that is further retained in mouse, rat, frog, fish, fungi and plants, as predicted by PrePS. This taxonomically widespread evolutionary conservation would rather indicate a relevance of the lipid anchor for the function of this protein, which is part of a family involved in transcriptional activation and chromatin formation, including histone binding [48] and nucleocytoplasmic shuttling [49]. The lack of the ability to be alternatively prenylated by GGT1 and, hence, being a unique FT substrate and putative FTI target, is also conserved in the other organisms, possibly pointing to the importance of the specific farnesyl anchor length. It should be noted that this protein is not predicted by the Prosite pattern PS00294 nor by the pattern derived from the rules of a few substrate-enzyme structures [23], but there exist other experimentally verified examples where the same CaaX box motif CKQQ has been shown to be farnesylated (yeast Pex19p [50] and human serine/threonine kinase 11 [51]).

While this paper was in preparation, farnesylation of the NAP1-like protein has been suggested experimentally through a special tagging and purification technique [52], giving support to the PrePS prediction. The same analysis, however, also suggests farnesylation of annexin A2 (GenBank accession number P07355) terminating in a CGGDD motif, which is not at all predicted by PrePS as it is mechanistically unlikely to be processed by farnesyltransferase. Another rather surprising prediction resulting from the tagging experiment is the farnesylation of Rab21 (Q9UL25), which has a double cysteine motif followed by three additional residues (CCSSG) which, at least formally, resembles a CaaX box. Rab proteins with CaaX boxes such as Rab5 (CCSN), Rab8 (CVLL/CSLL), Rab11 (CQNI) and Rab13 (CSLG) are usually modified by GGT2 *in vivo *[6,53,54] but Rab8 and Rab11 were shown also to be modified by GGT1 and FT *in vitro *[6,55]. PrePS predicts Rab21 to be geranylgeranylated by GGT2, but the prediction limit for farnesylation is not missed by far. The evOluation shows that the Rab21 orthologs in *Xenopus *(GenBank AAH60498.1) and *Drosophila *(AAH60498.1) share the double cysteines but their motif is different and shorter by one residue, pointing to a higher importance of the conservation of the cysteine doublet than the rest of the motif. The evOluation, furthermore, shows that Rab5 is the most closely related prenylated Rab-family member. Interestingly, both cysteines in the CCSN CaaX box motif of Rab5 were shown not only to be geranylgeranylated by GGT2 *in vivo *but are also required for proper localization and function of the GTPase [54]. Hence, a similar scenario for the two cysteines of the Rab21 prenylation motif cannot be excluded.

A complete analysis of large-scale predictions of prenylated proteins ranked by functional importance as estimated by evolutionary motif conservation and medical implications will be published in a follow-up work.

## Materials and methods

### Correlation of positional amino-acid frequencies with physical property scales

We identified physicochemical requirements for each motif position by correlating 20-dimensional vectors filled with the positional frequencies of occurrence of the 20 amino-acid types in the carboxy-terminally aligned learning set with a library of over 650 amino-acid physical properties [20,21]. This has been done over a largest subset of the learning set with removed redundancy of greater than 40% identity in the last 30 positions (nonredundant learning set = nrLS) and over positional vectors filled with frequencies derived from the profile (= prof) that has been corrected for redundancy with the position-specific independent counts (PSIC) method [56]. Such correlations have been estimated previously [12] to be significant for confidence levels α = 0.0025 and α = 0.001 if the values are greater than 0.62 and 0.7, respectively.

### Fisher criterion to find interpositional correlations

The Fisher ratio *F *of the sum of variances of single positions with the variance over multiple positions for pairs and triplets of positions is calculated, allowing gaps of up to two residues between pairs.



The *F *values for probabilities *p *= 0.05 are taken as thresholds for evaluating significance of interdependence of physical properties between motif positions. These are 1.288 for the FT and 1.355 for the GGT1 nonredundant learning set, respectively.

### Enzyme-specific binding pocket residue conservation

**Figure 6:**
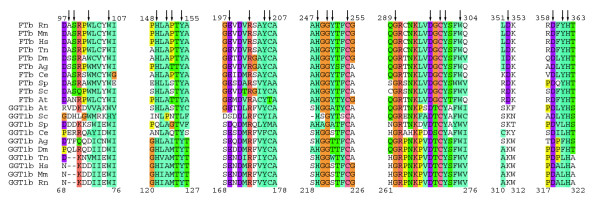
Alignment of FT and GGT1 beta subunits (FTb, GGT1b) in the regions of binding-pocket residues (marked with arrow) using ClustalX [57]. Residue ranges shown above and below correspond to the numbering in the PDB structures of rat FT beta (PDB 1D8D) and GGT1 beta (PDB 1N4Q), respectively. Accession numbers are as follows (GenBank unless indicated otherwise): Hs (*Homo sapiens*) FTb, NP_002019; GGT1b, NP_005014; Mm (*Mus musculus*) NP_666039; NP_766215; Rn (*Rattus norvegicus*) PDB 1D8D; 1N4Q; Tn (*Tetraodon nigroviridis*) CAG09215; CAF904630; Dm (*Drosophila melanogaster*) NP_650540; NP_525100; Ag (*Anopheles gambiae*) XP_321357; XP_317045; Ce (*Caenorhabditis elegans*) NP_506580; NP_496848; At (*Arabidopsis thaliana*) NP_198844; NP_181487; Sp (*Schizosaccharomyces pombe*) NP_594251; NP_594142; Sc (*Saccharomyces cerevisiae*) P22007; NP_011360. Standard ClustalX coloring (according to conserved amino acid type).

To identify regions in the FT and GGT1 binding pockets that are characteristic for the respective enzyme, we analyzed the pattern of residue conservation in vicinity to the CaaX substrate peptide and the prenylpyrophosphate (Figures 2 and 5). Alignments were created with FT and GGT1 beta subunits of a diverse subset of organisms (see legend of Figure 6) using ClustalX [57]. The conservation of alignment positions was measured using al2co [58] with the Henikoff-weighted variance-based options. The enzyme-specific conservation was calculated as conservation values in the FT or GGT1 alignments minus conservation values in a joined FT+GGT1 alignment (Figure 6) and then mapped to the binding pocket surface using a customized script in Swiss-Pdb Viewer [59]. Color transitions from white to yellow to red represent increasing conservation difference, where red signifies the highest enzyme-specific conservation. White parts are not characteristically different between FT and GGT1, but might also be well conserved between the two enzymes. As the observed differences in the binding pocket conservation relate to features of the enzyme and not the substrates, this information could not be taken directly into account in our scoring scheme. Indirectly, however, the resulting image mirrors the requirement for conservation also for the substrate peptides and, thereby, confirms the relative importance of motif positions as estimated by the Shannon entropy (see below).

### Prediction score function

Construction and calculation of the scoring function essentially follows the methodologies [7,16,60] applied to the prediction of GPI anchors [8], myristoylation [9] and PTS1 peroxisomal targeting [10] and is summarized shortly below with emphasis on additions and problem-specific variations.

The composite score function consists of a profile and physical property term.

***S ***= ***S***_profile _+ ***S***_ppt _    (2)

### The profile term *S*_profile_

The profile matrix is calculated using the PSIC algorithm to account for redundancy in the learning set. The frequency of occurrence *f*(*a*,*i*) of an amino acid type *a *at a given alignment position *i *is down-weighted proportionally to the number of other positions that are identical in sequences sharing *a *at *i*, resulting in subscores *S*_PSIC_(*a*,*i*) representing the natural logarithms of these redundancy-corrected frequencies. These are summed over the respective regions (+3, +2, +1 and -1 to -11)



before they enter *S*_profile_:



*α*_profile _is a normalization factor to compensate for differing lengths of the regions and is derived from:



*C*_region _is a relative weighting of motif regions. We propose a rational basis for its approximated computation. *C*_region _is derived from the average Shannon entropies of the regions in the alignment of the nonredundant learning set sequences. Shannon's information theoretic entropy has already previously been investigated as measurement of sequence variability in alignment positions and residue conservation [61]. We calculate the average of the exponential of the negative Shannon entropy as conservation measurement



where *n*_*a*,*i *_is the number of occurrences of amino-acid type *a *at the investigated position *i *of the region and *N *the total number of sequences in the alignment. Only amino-acid types that occur at least once (*n*_*a*,*i *_≥ 1) are included in the sum. In order to avoid overly precise values, the final values are rounded up to the last decimal that showed variation in the conservation measure when comparing the small learning set based on Swiss-Prot annotation with the larger learning set described in this work. The position of the absolutely conserved cysteine in the CaaX box would have the maximum conservation value of 1. Table 4 lists *C*_region _for the regions +1, +2, +3 and -11 to -1.

### The physical property term *S*_ppt_

The primary role of the physical property term is to exclude query sequences that do not fit into the determined physical property profile of the motif (see Figure 1). Hence, it does not add positive scores for compliance but only penalizes deviations from physical property requirements of the motif. These requirements have been identified as described above (listed in Tables 1 and 2; including rationale for inclusion into *S*_ppt_) and the biophysical meaning discussed earlier.

As an example, a penalty for a physical property *P *of the query being greater than the average physical property *P*_*j *_over the nonredundant learning set (with *σ*_*j *_being the corresponding standard deviation of a Gauss-like distribution) can be defined as follows:



The natural logarithms (to be comparable to the profile score) of these single terms enter *S*_ppt _as a sum with a term-specific weighting factor *α*_*j *_that emphasizes varying importance of the single terms.



As part of *S*_ppt_, the FT and GGT1 score functions also include a penalty for query proteins when the carboxy terminus appears inaccessible as a result of structural constraints. For example, helices with strongly hydrophobic sides are either buried in the structure or fold back to the protein core and reduce the flexibility and accessibility of the carboxy terminus. We recognize these if hydrophobic residues (LIVMFYW) appear in patterns like i,i+3,i+6 (hXXhXXh) or i,i+3,i+7 (hXXhXXXh) or i,i+4,i+7 (hXXXhXXh) within 20 residues preceding the cysteine of the CaaX box.

### Cross-validation (jackknife) tests

We have performed three jackknife tests to validate the robustness of our predictors. First, cross-validation over the whole scoring function (*S*_profile _+ *S*_ppt _recalculated after removal of the entry to be validated from the learning set) resulted in sensitivities of 99.4% for FT and 99.8% for GGT1, respectively. This generally indicates that the learning sets are large enough for the method not to suffer from removal of individual entries and, hence it should also be able to find valid motifs not yet included in the learning set. At the same time, we acknowledge that the similarity of sequences within homologous groups in the learning set influence the above estimated sensitivity. However, we emphasize that the values calculated for the profile matrix vary even when only one sequence from a group of homologs is left out, since the method used for profile extraction [56] does take into account such redundancy with both sequence- and position-specific weightings.

To address the role of homologous sequences remaining in the learning set for jackknife tests, we have extended the cross-validation to the more stringent case where not only the sequence to be predicted is excluded from the learning procedure but also its close homologs (estimated by a threshold of 40% sequence identity over the 30 carboxy-terminal residues). We find that in the case of the predictor for GGT1, the sensitivity of 98.6% is very close to the first jackknife test. For the FT predictor, a sensitivity of 92.6% is obtained in this test, which is only slightly lower than in the other jackknife procedures. The decrease can be almost exclusively attributed to the large group of highly similar sequences of hepatitis delta antigen that all share the uncommon motif CRPQ with both arginine at position +1 and proline at +2 being rather exceptional amino acids. Hence, the CRPQ sequences remain below the prediction threshold in the jackknife test if no example of this motif is present in the learning set. When leaving the CRPQ group out of the cross-validation, the FT predictor is calculated to have a sensitivity of 97.9%.

A third cross-validation test aims to elucidate whether the 39 parameters (13 terms with weightings, averages and variances, see Equations 7 and 8) introduced through the physical property terms in *S*_ppt _are overfitting the learning data. To exclude bias through similar sequences in homologous groups, the test was executed only over the learning set after removing redundancy (see section on correlation analysis above). *S*_ppt _alone is recalculated after removal of the entry to be validated from the parameter calculation procedure. The obtained sensitivities of 100% for FT and 99.8% for GGT1 indicate that the parameterizations of physical property terms in *S*_ppt _are not overfitting the learning data.

### Probability of false-positive prediction

To estimate probabilities of false-positive prediction, a set of sequences had to be defined whose carboxy-terminal amino-acid pattern should not be subject to selection for a valid CaaX prenylation motif. This is fulfilled for sequences in the NCBI nonredundant database lacking a cysteine at the fourth position from the carboxy terminus. Hence, any compliance with motif restrictions of such sequences apart from the fourth last position could only be attributed to random or at least prenylation-independent appearance. Following earlier experience with GPI- and myristoyl-anchor prediction [8,9], a polynomial extreme-value distribution function has been fitted to the scores obtained for the described sequence set when ignoring the requirement of a CaaX box cysteine in the procedure. The probability of obtaining a score *S *greater than a threshold score *S*_*th *_can be formulated as follows:





A polynom of the sixth degree was used to improve the residual fit. Polynoms with degrees higher than six would not result in an increase of the relative improvement of the residual. The probabilities of false-positive prediction for scores at the prediction threshold are extrapolated to 6.3% for FT and 1.2% for GGT1 for sequences that contain a cysteine at the fourth-last position (canonical CaaX box). Given that appearance of a cysteine at this position is rare in databases (1.7%), the independent general probabilities of false-positive prediction by FT and GGT1 for all protein sequences are as low as 0.11% and 0.02%, respectively. This corresponds to specificities of 99.89% and 99.98%.

Since the subcellular context of a protein can be relevant to judging the likelihood of *in vivo *prenylation when a corresponding motif has been predicted, we also tried to estimate probabilities of false-positive prediction for differently localized subsets of proteins. These subsets were retrieved from the Swiss-Prot database [22] and assigned according to the following keywords in the 'Subcellular Location' comment lines: 'cytoplasmic' (24,284), 'nuclear' (9800), 'membrane' (24,823) and 'extracellular' (509). Parentheses indicate the number of unambiguously annotated examples per subset. Again, only proteins lacking a -CXXX motif were taken into account, to ensure prenylation-independent selection of carboxy-terminal amino-acid residues. Although there appear to be some subcellular localization-specific fluctuations of the probabilities of false-positive prediction (partly due to limited or differing subset sizes), the relative advantages among the methods evaluated seem to remain stable (see Table 3).

## Figures and Tables

**Table 1:** Physical property terms in the FT scoring function

Property	Position	Rationale	Explanation
ARGP820103 [62]	+3	Corr = 0.7(nrLS)	Membrane-buried preference, lipid contact when entering binding pocket
logPREN_CKQX_FT [15]	+3	Corr = -0.72(nrLS)	Kinetic measurement, relative unprocessed FPP amounts with tetrapeptide CKQX
CHOC760101 [63]	+1 to +3	Fisher = 1.3	Side chain volume
ZVEL_CHARG [64]	+1 to +3	LS composition	General charge penalty
ZVEL_CHNEG [64]	+1 to +3	LS composition	Special negative charge penalty
WERD780102 [65]	+1 and +3	Fisher = 1.51	Hydrophobicity compensation for inside preference
ZVEL_ALI_1 [64]	+1 and +2	+2: Corr = 0.85(prof) +1: continuing deviation from Uniref50 average	Amino-acid property: aliphatic
LIFS790102 [66]	+1 and +2	+2: Correlation = 0.76(prof) +1: continuing deviation from Uniref50 average	Preference for extended conformations
ZVEL_TINY_ [64]	-1	Corr = 0.68(prof)	Size, bulkiness
MOBILITY_2 [21]	-1	Corr = 0.61(nrLS)	Side chain mobility
VINM940101 [67]	-11 to -1	-2: Corr = 0.72(prof) -3: Corr = 0.75(prof) -4: Corr = 0.78(nrLS) -5: Corr = 0.82(nrLS) -6: Corr = 0.84(nrLS) -7: Corr = 0.79(nrLS) -8: Corr = 0.74(prof) -9: Corr = 0.82(nrLS) -10: Corr = 0.84(nrLS) -11: Corr = 0.79(nrLS) Rest: continuing deviation from Uniref50 average	Normalized flexibility average
KRIW790102 [68]	-11 to -1	-2: Corr = 0.76(prof) -6: Corr = 0.83(nrLS) -7: Corr = 0.83(nrLS) -8: Corr = 0.76(prof) Rest: continuing deviation from Uniref50 average	Fraction of site occupied with water
Buried helix (see Materials and methods)	-20 to -1	Remove false positives	Helix with strongly hydrophobic sides folds back to protein core and reduces flexibility and accessibility of C-terminus

Corr, correlation; LS, learning set; nrLS, nonredundant; prof, profile.

**Table 2:** Physical property terms in the GGT1 scoring function

Property	Position	Rationale	Explanation
ARGP820103 [62]	+3	Corr = 0.8(prof)	Membrane-buried preference, lipid contact when entering binding pocket
LEVM760105 [69]	+1 to +3	Fisher = 1.36	Size limitation (radius of gyration of side-chain)
YUTK870101 [70]	+1 to +3	Fisher = 1.38	Hydrophobicity compensation (Unfolding Gibbs energy in water, pH7.0)
ZVEL_CHARG [64]	+1 to +3	LS composition	General charge penalty
ZVEL_CHNEG [64]	+1 to +3	LS composition	Special negative charge penalty
ZVEL_ALI_1 [64]	+1 and +2	+2: Corr = 0.87(prof) +1: continuing deviation from Uniref50 average	Amino-acid property: aliphatic
LIFS790102 [66]	+1 and +2	+2: Corr = 0.77(prof) +1: continuing deviation from Uniref50 average	Preference for extended conformations
FAUJ880101 [71]	-1 and +2	Fisher = 1.52	Size, bulkiness (residues although 10 Å apart, face to same side of base pair)
FINA910103 [72]	-1	Corr = 0.75(prof)	Helix termination (for example, K, S favored, D,E,L,I,V disfavored)
KARP850103 [73]	-7 to-1	-1: Corr = 0.69(prof) -2: Corr = 0.70(prof) -3: Corr = 0.71(prof) -4: Corr = 0.74(nrLS) -5: Corr = 0.75(prof) -6: Corr = 0.70(nrLS) -7: Corr = 0.78(nrLS)	Flexibility (GGT1 lysine preference)
VINM940101 [67]	-11 to -1	-4: Corr = 0.72(prof) -5: Corr = 0.82(prof) -6: Corr = 0.84(nrLS) -7: Corr = 0.75(nrLS) -8: Corr = 0.77(nrLS) -9: Corr = 0.68(prof) -10: Corr = 0.86(prof) Rest: continuing deviation from Uniref50 average	Normalized flexibility average
KRIW790102 [68]	-11 to -1	-3: Corr = 0.70(prof) -4: Corr = 0.73(prof) -5: Corr = 0.84(prof) -6: Corr = 0.81(prof) -7: Corr = 0.83(nrLS) -8: Corr = 0.85(nrLS) -9: Corr = 0.76(prof) -10: Corr = 0.86(prof) Rest: continuing deviation from Uniref50 average	Fraction of site occupied with water
Buried helix (see Materials and methods)	-20 to -1	Remove false positives	Helix with strongly hydrophobic sides folds back to protein core and reduces flexibility and accessibility of carboxy terminus

**Table 3:** Comparison of prediction performances

	FT			GGT1		
		
	Prosite PS00294	Beese, Casey and colleagues' rules	PrePS FT	Prosite PS00294	Beese, Casey and colleagues' rules	PrePS GGT1
Sensitivity I	85%*	*72%*	**100%**	95%*	*67%*	**100%**
Sensitivity II	NA	NA	**92.6/97.9%**^†^	NA	NA	**98.6%**
Probability of false positive prediction (POFP) for -CXXX motifs (GenBank sequences)	17.1%*	9.9%	**6.3%**	17.1%*	10.0%	**1.2%**
POFP -CXXX 'cytoplasmic'^‡^	18.2%*	8.9%	**5.1%**	18.2%*	8.6%	**1.4%**
POFP -CXXX 'nuclear'^‡^	13.9%*	10.5%	**5.5%**	13.9%*	9.6%	**1.1%**
POFP -CXXX 'membrane'^‡^	17.5%*	10.3%	**3.8%**	17.5%*	12.0%	**0.8%**
POFP --CXXX 'extracellular'^$^	8.6%*	7.9%	**3.3%**	8.6%*	*9.0%*	**0.2%**
Overall probability of false positive prediction (GenBank sequences, assuming 1.7% with -CXXX)	0.29%*	0.16%	**0.11%**	0.29%*	0.17%	**0.02%**

*Prosite pattern PS00294 does not distinguish between prenylation by FT and GGT1.

^†^Sensitivity rises to 97.9% when the exceptional motif CRPQ of hepatitis delta antigen is removed. ^‡^For details see Materials and methods. Sensitivity I is the rate of finding known substrates from described learning set = self-consistency. Sensitivity II is the rate of finding known substrates after their exclusion (including homologs) from the learning set = cross-validation (see Materials and methods). Probabilities of false-positive predictions (POFP) complement the specificities to 100% (Specificity = 100 - POFP). The first listed POFP estimates the rates of false positives among query proteins that have a canonical -CXXX motif (which corresponds to 1.7% of all sequences). Below are estimations of POFPs for subsets of Swiss-Prot proteins that differ in their annotated subcellular localization (see Materials and methods). The final POFP is the estimate for false-positive predictions for all sequences (for example, when analyzing complete proteomes or large databases), independent of existence of a -CXXX motif. Formatting signifies: best (bold), intermediate (plain text), worst (italic) performance.

**Table 4:** Relative weightings of motif positions in profile term

Position(s)	FT	GGT1
-11 to -1	0.07	0.07
+1	0.15	0.14
+2	0.29	0.41
+3	0.17	0.42
